# Supplementary material for: Chemotherapy-Induced Changes in the Lung Microenvironment: The Role of MMP-2 in Facilitating Intravascular Arrest of Breast Cancer Cells
Source: Int J Mol Sci. 2021 Sep 24;22(19):10280. doi: 10.3390/ijms221910280 (PMC8508901; doi:10.3390/ijms221910280)
Supplement: Supplementary file 1 [file ijms-22-10280-s001.zip › ijms-1371780-supplementary.pdf]

Supplementary Information for:

Chemotherapy-induced changes in the lung microenvironment: the role of MMP-2 in facilitating intravascular arrest of breast cancer cells

Justin D. Middleton[1,2], Subhakeertana Sivakumar[1], Tsonwin Hai[1,2*]

[1] Department of Biological Chemistry and Pharmacology
[2] Molecular, Cellular, and Developmental Biology Program

*Correspondence: Tsonwin Hai

Email: hai.2@osu.edu

This PDF file includes:

Figures S1 to S7

Tables S1 to S2

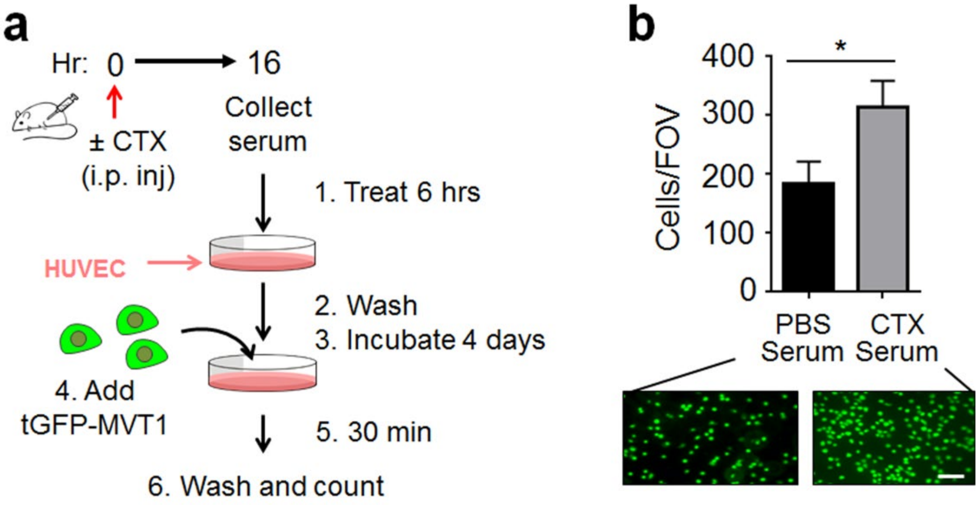

Figure S1. CTX increases the adhesiveness of human umbilical vein endothelial cells (HUVECs). *(a)* A schematic of the experiment, which is the same as that in Figure 3a, except using HUVEC monolayers (rather than mLEC) and a 4-day incubation (rather than 2-day). *(b)* Average cell count per field of view (FOV) from nine images obtained per well (N=12-13 from 3 independent experiments). Scale bar: 50 µm. Bars indicate mean ± SEM; Student's t-test; $*P < 0.05$.

a

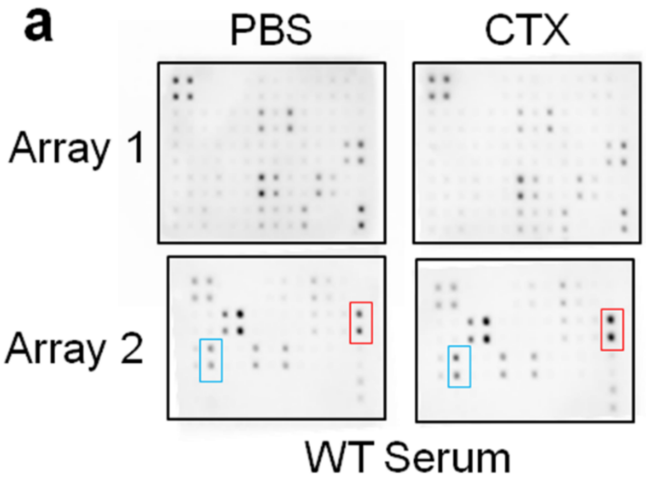

b

Array 1

POS	POS	NEG	NEG	Blank	Axl	CXCL13	CD30L	CD30	CD40	CRG-2	CCL27	CXCL16	CCL11
POS	POS	NEG	NEG	Blank	Axl	CXCL13	CD30L	CD30	CD40	CRG-2	CCL27	CXCL16	CCL11
CCL24	FasL	CX2CL1	G-CSF	GM-CSF	IFNg	IGFBP-3	IGFBP-5	IGFBP-6	IL-1a	IL-1b	IL-2	IL-3	IL-3R
CCL24	FasL	CX2CL1	G-CSF	GM-CSF	IFNg	IGFBP-3	IGFBP-5	IGFBP-6	IL-1a	IL-1b	IL-2	IL-3	IL-3R
IL-4	IL-5	IL-6	IL-9	IL-10	IL-12 p40/p70	IL-12 p70	IL-13	IL-17A	CXCL1	LeptinR	Leptin	LIX	L-sel
IL-4	IL-5	IL-6	IL-9	IL-10	IL-12 p40/p70	IL-12 p70	IL-13	IL-17A	CXCL1	LeptinR	Leptin	LIX	L-sel
Ltn	MCP-1	MCP-5	M-CSF	CXCL9	MIP-1a	MIP-1g	MIP-2	MIP-3b	MIP-3a	PF-4	P-sel	CCL5	SCF
Ltn	MCP-1	MCP-5	M-CSF	CXCL9	MIP-1a	MIP-1g	MIP-2	MIP-3b	MIP-3a	PF-4	P-sel	CCL5	SCF
SDF-1a	CCL17	CCL1	CCL25	TIMP-1	TNFa	TNFR1	TNFR2	TPO	VCAM-1	VEGFA	Blank	Blank	POS
SDF-1a	CCL17	CCL1	CCL25	TIMP-1	TNFa	TNFR1	TNFR2	TPO	VCAM-1	VEGFA	Blank	Blank	POS

Array 2

POS	POS	NEG	NEG	Blank	bFGF	CD26	Dtk	E-sel	FcGRIIb	Flt3	GITR
POS	POS	NEG	NEG	Blank	bFGF	CD26	Dtk	E-sel	FcGRIIb	Flt3	GITR
HGFR	ICAM1	IGFBP-2	IGF-1	IGF-2	IL-15	IL-17 RB	IL-7	CXCL11	CXCL15	CCL22	MMP2
HGFR	ICAM1	IGFBP-2	IGF-1	IGF-2	IL-15	IL-17 RB	IL-7	CXCL11	CXCL15	CCL22	MMP2
MMP3	SPP1	OPG	Pro-MMP9	Resistin	Shh-N	CXCL7	TIMP2	TRANCE	TROY	TSLP	VEGFR1
MMP3	SPP1	OPG	Pro-MMP9	Resistin	Shh-N	CXCL7	TIMP2	TRANCE	TROY	TSLP	VEGFR1
VEGFR2	VEGFR3	VEGFD	Blank	Blank	Blank	Blank	Blank	Blank	Blank	Blank	POS
VEGFR2	VEGFR3	VEGFD	Blank	Blank	Blank	Blank	Blank	Blank	Blank	Blank	POS

Figure S2. Protein arrays comparing WT sera. *(a)* Images of the two arrays comparing sera from WT mice at 16 hours after treatment with CTX or PBS. Red box: MMP-2, blue box: OPN. *(b)* A map of the proteins detected in the arrays.

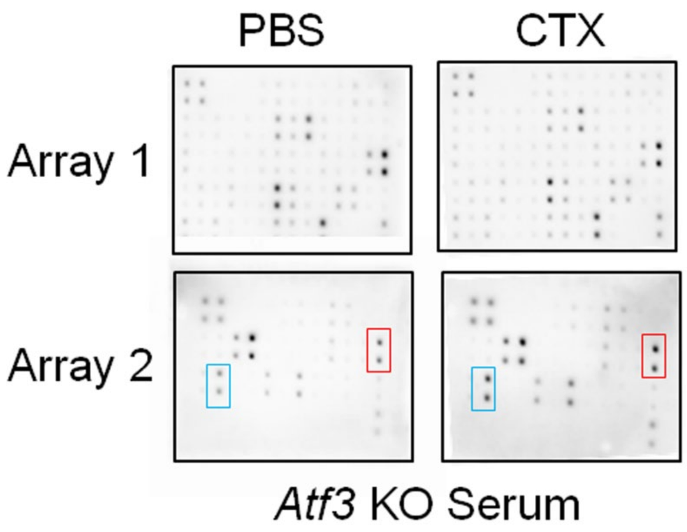

Figure S3. Protein arrays comparing *Atf3* KO sera. Images of the two arrays comparing sera from *Atf3* KO mice at 16 hours after treatment with CTX or PBS. Red box: MMP-2, blue box: OPN.

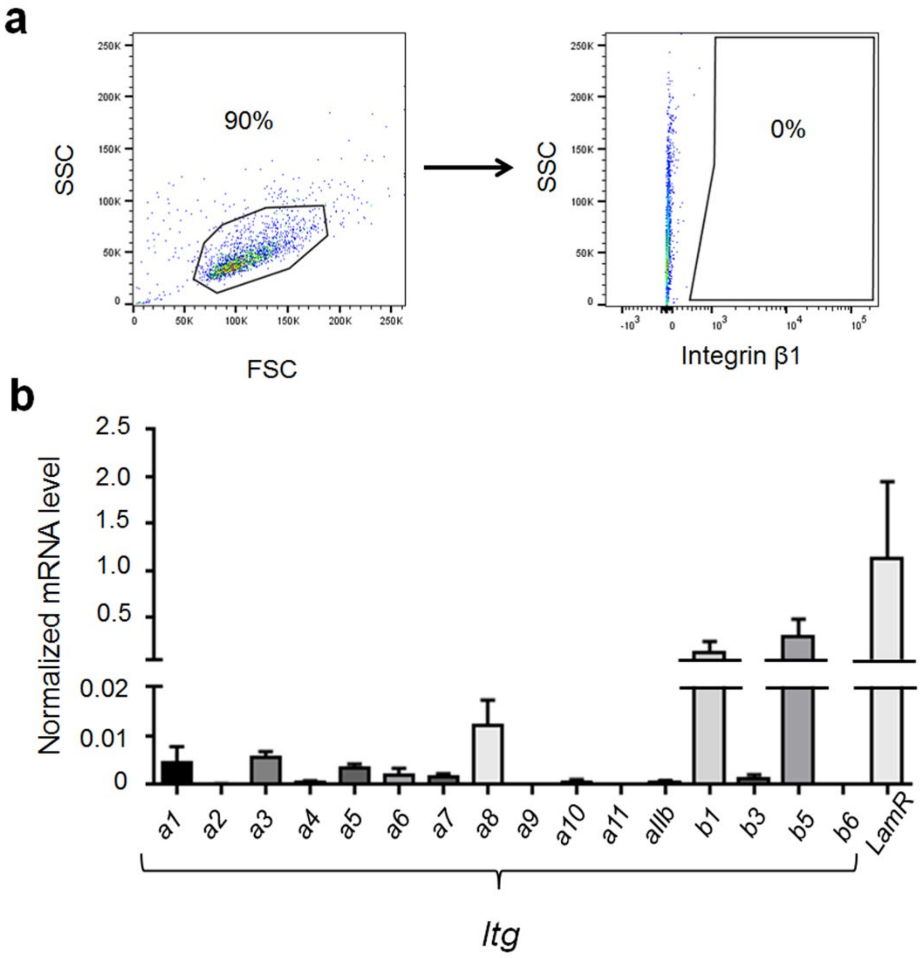

Figure S4: High levels of integrin β1 and laminin receptor (<u>*LamR*</u>) mRNA are present in tGFP-Met1 cells. *(a)* A representative image of the initial gating of tGFP-MVT1 cancer cells to remove debris and cell aggregates, followed by a representative image of positive integrin β1signal gating as determined using fluorescence minus one control. *(b)* mRNA levels *LamR* and the indicated integrins (*Itg*) in tGFP-Met1 cells were analyzed by reverse transcriptase coupled with quantitative polymerase chain reaction (RT-qPCR). Signals were normalized against glyceraldehyde 3-phosphate dehydrogenase (*Gapdh*) (from 3 independent experiments). Bars indicate mean ± SEM.

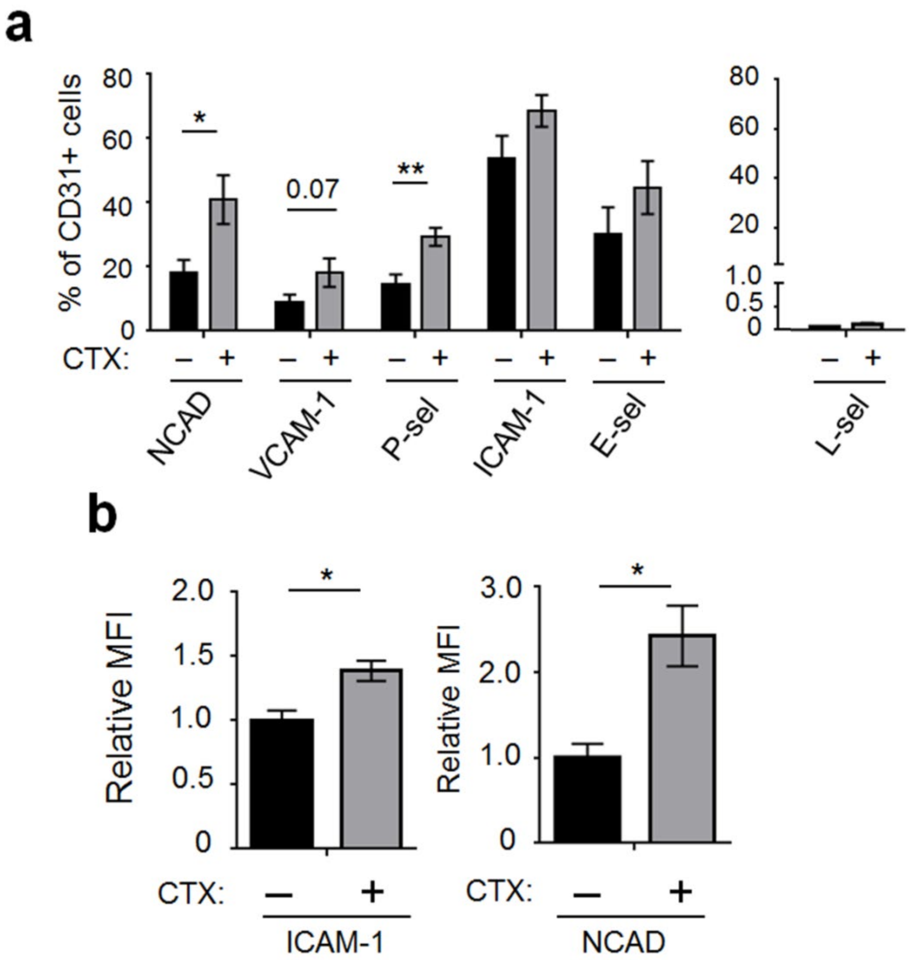

Figure S5. CTX increases the presence of cell adhesion molecules (CAMs) on the surface of lung endothelial cells. *(a)* Percentage (%) of $CD31^+$ cells from the lung positive for the indicated CAMs (N=9-14 mice from 3 independent experiments). NCAD: N-cadherin; VCAM-1: vascular cell adhesion molecule 1; P-sel: P-selectin; ICAM-1: Intercellular adhesion molecule 1; E-sel: E-selectin; L-sel: L-selectin. *(b)* Relative mean fluorescent intensity (MFI) of the indicated CAMs on $CD31^+$ lung cells (N=9-14 mice from 3 independent experiments). Bars indicate mean ± SEM; Student's t test; $*P < 0.05$, $**P < 0.01$.

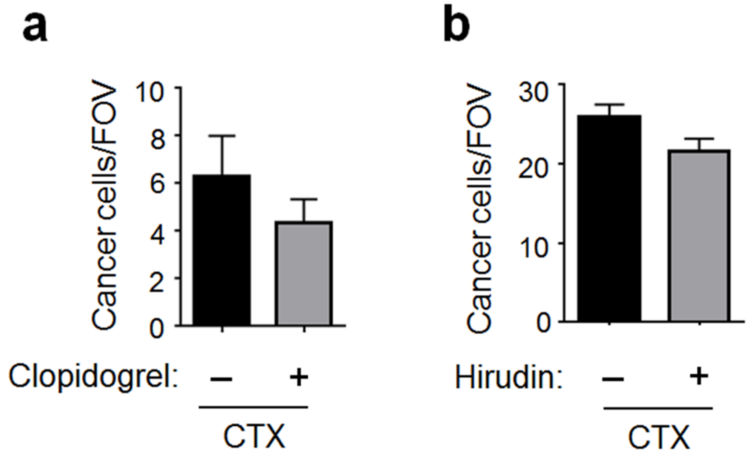

Figure S6: Inhibition of platelet activation did not affect intravascular cancer cell arrest. *(a)* Mice were pre-treated with CTX at four days before cancer cell injection (tGFP-MVT1) as in Figure 1, with the addition of intraperitoneal injection of clopidogrel (+) at 10 mg/kg or vehcle (-)18 hours before and 2 hours after CTX pre-treatment. Lungs were collected at 9 hours after cancer cell injection for analysis (N=3-5 mice/group). *(b)* Mice were pre-treated with CTX at four days before cancer cell injection (tGFP-MVT1) as in Figure 1, with the addition of intravenous injection of hirudin (+) at 10 mg/kg or vehicle (-) 20 minutes prior to cancer cell injection. Lungs were collected at 3 hours after cancer cell injection for analysis (N=4 mice/group). Bars indicate ± SEM; Student's t-test. No statistical significance was observed between the vehicle control (-) and treatment (+) groups for both drugs.

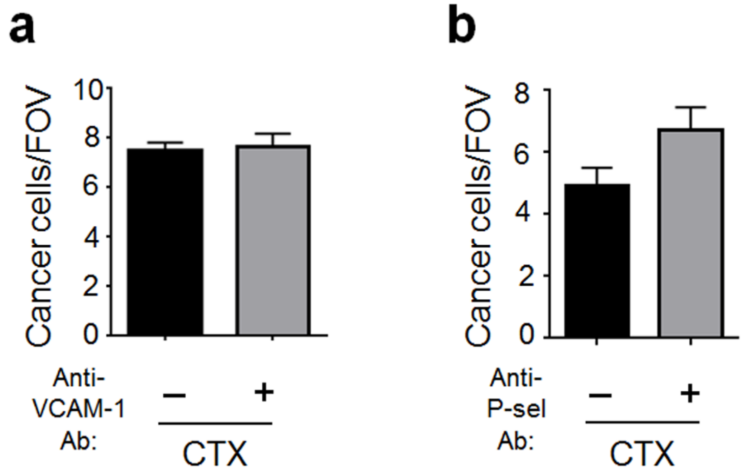

Figure S7. Blocking antibodies against VCAM-1 and P-sel did not affect intravascular cancer cell arrest. *(a)* Mice were pre-treated with CTX at four days before cancer cell injection (tGFP-MVT1) as in Figure 1, with the addition of intravenous injection of 100 µg anti-VCAM-1 antibody (Ab, +) or 100 µg isotype IgG antibody (-) at 2 hours prior to cancer cell injection. Lungs were collected at 3 hours after cancer cell injection for analysis (N=3-4 mice/group). *(b)* Same as in panel (a) except 25 µg of the anti-P-sel antibody was used. Lungs were collected at 3 hours after cancer cell injection (N=3 mice/group). Bars indicate ± SEM; Student's t-test. No statistical significance was observed between the control (-) and treatment (+) groups for both antibodies.

Table S1. Primers used in this study

Target	Forward	Reverse
Itga1	5'-TATCCTCCTGAGCGCCTTT-3'	5'-TGGCCTTTTGAAGAATCCAA-3'
Itga2	5'-GGTTCTGCAGGATAGAAACCA-3'	5'-TGGACACCGTCTTCAGTAGAAA-3'
Itga3	5’-GCGGAAGGACTGGGATTTAT-3’	5’-GATGATGTCCGTGGGATGTAG-3’
Itga4	5′-AATTGGACCAAGTGAGGGACAA-3′	5′-TCGCTAGATCCATACACAAATGAAGT-3′
Itga5	5'-TCG GAG CAA CAG TTC GGG-3'	5'-GTGGAGCACATGCCAAGATG-3'
Itga6	5’-ACCTCGGCACAGCAACCTTGA-3’	5’-ACGCTGCAGTTGAGAGTCTGGT-3’
Itga7	5'-GATCGTCCGAGCCAACATCACA-3'	5'-CTAACAGCCCAGCCAGCACT-3'
Itga8	5'-TCAAGGCGAGGAACAGCAA-3'	5'-CCTTGGGAACCCGATGGT-3'
Itga9	5'-ATGACGGGTTCCCAGATG-3'	5'-TGTAGACTGCGCCAGCAA-3'
Itga10	5'-GGCTCCAACAGT ATCTATCC-3'	5'-TGCTCTCACAACTTCTTCC-3'
Itga11	5'-GGACTTCTTCACCGACCAGG-3'	5'-CAGCCTCAGGTTGCAGATGA-3'
ItgaIIb	5'-ACATTGAGGGCTTTGAGAGGCT-3'	5'-TTGCCACAGGCAACATCACG-3'
Itgav	5’-TGAAGTCTTTCGGCTCTGCG-3’	5’-CATCCTGGAGGACGTGCTGG-3’
Itgb1	5’-GCAGGTGTCGTGTTTGTGAATGCT-3’	5’-ACAAGTTGGCCCTTGAAACTTGGG-3’
Itgb3	5′-GGACACAGCCAACAACCCAC-3′	5′-AGGAGGCATTCTGGGACAAAG-3′
Itgb5	5'-TGTTCAGCTACACAGAACTGCCCA-3'	5'-TTTGGAACTTGGCAAACTCTCGGC-3'
Itgb6	5’-AGATGGACTTGTTCTTGGGTG-3’	5’-GACAGCAAGCTGGCAGGCATTG-3’
LamR	5'-GGTGGCACCAACCTTGACTTTC-3'	5'-GTCAGCAGGATTCTCGATGGCA-3'
Gapdh	5’-CAA CGG GAA GCC CAT CA-3’	5’-CGG CCT CAC CCC ATT T-3’

Table S2. Antibodies used in this study

**Name/Property**	**Assay**	**Dilution/Amount**	**Item #**	**Company**
anti-CD16/CD32 Fc	Flow cytometry	0.5 μg/100 μl	14-0161-86	Invitrogen
anti-CD31	Flow cytometry	0.125 μg/100 μl	12-0311-82	eBioscience
anti-ITGB1	Flow cytometry	0.125 μg/100 μl	47-0291-82	Invitrogen
anti-P-sel	Blocking	25 μg/mouse	12-0626-82	ThermoFisher
anti-tGFP	IF	1:2000	PA5-22688	Invitrogen
anti-VCAM-1	Blocking	100 μg/mouse	NBP1-26587	Novus
CD31 MicroBeads	MACS	10 μl/100 μl	130-097-418	Miltenyi Biotec
Mouse IgG	Blocking	25 μg/mouse	12-4714-82	Invitrogen
Rat IgG	Blocking	25 μg/mouse	MAB005	Novus

IF: Immunofluorescence
